# 

**Published:** 1883-06

**Authors:** 


### CONTENTS.

### COMMUNICATIONS.

Etiology and Pathology of Dental Caries and Treatment of Pulps.. 255
On the Functions of the Nerves of Taste........ 261
On Nicotine and its Action on the Teeth........ 268
A Proposed Dental Reform........ 293
The Late Dr. Marshall H. Webb........ 295

### PROCEEDINGS.

Minutes of the Michigan Dental Association........ 273

### SELECTIONS.

On the Conservative Treatment of Exposed Pulp........ 282
On Regulating Teeth........ 285
A Tooth at Birth........ 287
Who Discovered the Anæsthetic Properties of Ether........ 288
The Struggle for a Living........ 288
Mixed Anæsthesia by Ether, Morphine, and Atropine........ 289
Leeches........ 291

### BIOGRAPHICAL.

Biography of Dr. T. B. Hamlin........ 296

### EDITORIAL.

Indiana State Dental Meeting........ 301
Kentucky State Dental Association........ 303
Hymenial........ 303
Removal........ 303
A New Rubber Clamp........ 304
Educational Association........ 304
Meeting—Mad River........ 305
Obituary........ 305
A Suggestion—Journalistic........ 306

